# Progress in Obstetrics

**Published:** 1898-04-16

**Authors:** 


### Progress in Obstetrics.

(*Continued from page 32.*)

**Pregnancy Complicated with Cancer of Uterus.**—Borche[4] considers that it is desirable to allow pregnancy to reach term, and condemns the methods of intervention by artificial abortion, amputation of the cervix, and total hysterectomy during pregnancy; the first because the diagnosis of pregnancy is very difficult to make with certainty before the fourth month, and abortion to be of service must be produced before this; the second because as a rule the result is unfavourable and the relief only temporary, while there is great danger to the fœtus; while the third method, if performed by supra-vaginal amputation after Cæsarian section, necessitates the dealing with tissue probably diseased already, and the rapid spread of the disease during pregnancy renders the infection of neighbouring structures almost certain. Many of these patients have a natural delivery; if dilatation is difficult it should be assisted by Champetier de Ribes' bag, Tarnier's dilator; if dilatation is incomplete, but sufficiently advanced for the operation to effect natural delivery, and if the walls of the cervix are not very thick, by multiple incisions into the cervix. Forceps may be applied if sufficient dilatation is present, and craniotomy and cephalotripsy performed if the child is dead. If dilatation cannot take place owing to the growth encircling the os, Cæsarian section is indicated, and should be performed in such cases at term, but before the commencement of labour. The uterus should not be removed, if this can be avoided, on account of the previously mentioned infection of neighbouring tissues.

**Vaginal Cœliotomy for Removal of Multiple Uterine Myomata.**—The conservative method of treatment of uterine fibroids by operation, with the removal of the tumours rather than the complete ablation of the organ, has of late gained ground, and appears to be, surgically speaking, a satisfactory operation in many cases in the hands of experienced operators. The preservation of the uterus and restoration of its normal function in such cases is of sufficient importance to render resort to such operations, where practicable, preferable to the various operations for complete removal of the uterus. Wendel and Bailey[5] describe fourteen cases in which they practised this operation with encouraging results. They consider that multiple myomata rarely remain stationary in growth, and usually develop more rapidly and cause more acute symptoms, even in those cases where they are of small size, than solitary myomata, which as a rule give rise to less acute symptoms. The writers advise operation and the early removal of even small multiple tumours in every case where prolonged observation shows growth, especially if extension into the broad ligament is noted, since the earlier the operation is performed the easier it will be. They consider that hysterectomy is called for if associated with the tumours suppurative endometritis is present, with removal of the appendages in addition if suppurative disease of the appendages exists. The preparatory treatment for the operation is the same as for hysterectomy; the lithotomy position is used, and the patient is anæsthatised. To exclude the anus from the field of operation, since it is almost impossible to secure asepsis otherwise, a rubber dam is used, which is stitched to the perineum and the patient's thighs; this dam has an aperture corresponding in size to the vulvar commissure, the perineum is retracted with a speculum, and the uterine cavity curetted and washed out, but not packed. Erosions are cauterised with the Paquelin cautery, and after flushing out the vagina the labia are separated by lateral retractors. If the tumours are situated on the posterior aspect of the uterus the posterior *cul de sac* is opened, and the posterior surface carefully explored through the pouch of Douglas; if on the anterior, the anterior fornix is laid open through a sagitto-coronal incision. The peritoneal openings should be close to the uterus, and extended well on to the broad ligaments. There should be a plentiful supply of fine volsella; long, sharp, and blunt-pointed tenotomes; long-handled, fine clamps; large and small Hagedorn's needles, threaded with medium chromicised catgut; curved and straight iridectomy scissors with long handles; and needle holders. The cervix is drawn down and the uterus carefully palpated between the fingers to detect the number and location of the tumours. The affected surface of the uterus can now be exposed and an incision made over the most prominent tumour, and the capsule stripped back. The tumour, if larger than a hazel nut, is grasped with a small volsellum and morcellated; if not, it is twisted out and the bed sutured with interrupted buried sutures drawn tight; if several tumours are contiguous the suturing is left until all have been removed. If the uterine cavity is opened the sutures must be carried through the wall without entering the cavity. Openings in the parametrium are drained with gauze strips brought out into the vagina. Adhesions, if present, are carefully separated.

The authors operated on fourteen cases, removing tumours varying in number in each case from two (smallest), to eleven (largest); the individual tumours ranged in size from that of a pea to a large orange-

Pregnancy occurred in two cases; one went to full term, the other aborted after criminal induction. Two of the fourteen were single and nulliparous. In all cases prolonged treatment by drugs, electricity, &c., had been tried without success.

The authors consider that the operation performed in this way is far preferable to the ventral route, and has the advantage of avoiding the psychopathic disturbances which so often follow complete hysterectomy, while the facilities for drainage and avoidance of handling the intestines render convalescence more rapid.

**The Obstruction of Labour by Ovarian Tumours in the Pelvis.**—This important complication is not so rare as is usually supposed, and requires to be dealt with promptly, because in many of the cases in which it exists its presence is unsuspected during pregnancy.

McKerron,[6] in a paper read before the Obstetrical Society of London, has collected 183 cases of this complication, and analysed the results of various methods of treatment. The alternatives are: (1) To leave the case to nature, assisted by forceps, turning, or craniotomy if necessary; (2) to attempt reposition by pushing the tumour up out of the pelvis; when this fails either (3) puncture or incision of the cyst; (4) Cæsarian section, or (5) abdominal or vaginal ovariotomy. He tabulates the various results of these methods of treatment as follows: Of 35 cases left to nature, 12 mothers died; of 41 cases treated by reposition of the tumour, 5 mothers died; of 43 cases treated by puncture or incision, 8 mothers died; of 17 cases treated by version, 6 mothers died; of 14 cases treated by forceps, 8 mothers died; of 18 cases treated by craniotomy, 8 mothers died; of 10 cases treated by Cæsarian section, 8 died; and of cases treated by ovariotomy 2 treated by abdominal ovariotomy were both successful, as also were 3 treated by vaginal ovariotomy, a total mortality of 49, or 26·7 per cent. This shows the different methods of treatment adopted and the mortality of each, from which, however, definite conclusions cannot be drawn, since the circumstances of the respective cases differ widely. The results of ovariotomy certainly seem the best, and those of Cæsarian section the worst, but the latter was performed in some, though not in all, cases on account of dense adhesions. The high mortality after reposition can be accounted for by the occurrence of twisting of the pedicle, a very probable accident, or rupture of the cyst into the peritoneal cavity.

McKerron concludes: (1) That where Cæsarian section is necessary the tumour should also be removed; (2) when the cyst contents are proved or strongly suspected to be infectious, or when the tumour has been subjected to long-continued pressure abdominal ovariotomy should be performed immediately, or a few hours after labour; (3) when the tumour has been subjected to considerable pressure, and is believed to be a dermoid, its removal should be effected at the end of the first week; (4) where reposition has been successful early in labour, or where puncture reveals the tumour to be a simple cyst, expectant treatment should be adopted, but the supervention of inflammatory symptoms should be at once followed by laparotomy. In the discussion which followed McKerron's paper the difference in treatment brought about by the introduction of antiseptics was strongly insisted upon, since the mortality of operations performed twenty years ago is no guide to that of those performed in the last five or ten years. It was considered that measures, such as the use of the forceps, craniotomy, and version should be discarded, as should tapping, while Cæsarian section should be seldom necessary, since delivery of the uterus through the abdominal incision usually allowed of the withdrawal of the tumour, after which labour could be terminated per vias naturales. The operation of vaginal incision of the cyst and the sewing of its edges to the edges of the vaginal incision would often allow of extra-peritoneal evacuation of its contents, and avoided the chief danger of tapping, which McKerron's paper showed to be attended with a mortality of about 50 per cent. This operation, however, should not be done if reposition could be effected, or if the attendant was a skilled ovariotomist. As a large number of the tumours causing this complication were dermoid cysts, it was not always certain that simple tapping by the vagina would effect a sufficient evacuation of their contents to allow of delivery being effected.

**Labour in Mature Primiparæ.**—The well-known difficulties occurring in mature primiparæ have been embodied in an instructive paper by De Konninck[7]. He refers to primiparæ married for some years, and relatively mature. He gives 30 as the earliest year coming under the term "maturity," and sets aside those rare cases where pregnancy has occurred for the first time at ages of 50 and over.

In many of the cases described the catamenia are found to have occurred for the first time later than usual, *e.g.*, at 20 and over in 10 per cent. Such accidents as abortion and ectopic gestation are relatively frequent, as are also the occurrence of renal disease and the presence of twins. Statistics show that lingering labour is even more common than is popularly supposed. He found that in 12 cases out of 17 noted by him labour lasted from 40 to 50 hours, the remaining five being still longer, and one exceeding 90 hours. Feebleness of uterine contractions is absolute from the beginning, even independently of any obstetric complication, and they are at the same time more painful. Much more physical and mental exhaustion is caused than in young primiparæ. The relative age of the uterus in one patient may be greater than in another, *i.e.*, the contractions in two cases of the same age, may correspond in one to those of a woman of maturer years, while in the other they may resemble those of a younger woman. This would seem to be comparable to the variations in the date of the menopause in different women. Obstetric operations to assist delivery are often required in these cases, and it is a curious fact that in these cases also a large excess—30 per cent.—of the children are males.

[4] Clinical Journal, March 2, 1898. [5] New York Med. Record, January 28, 1898. [6] Obstetrical Transactions, 1897, and B. M. J., January 15, 1898. [7] B. M. J., January 1, 1898.

